# BS Seeker: precise mapping for bisulfite sequencing

**DOI:** 10.1186/1471-2105-11-203

**Published:** 2010-04-23

**Authors:** Pao-Yang Chen, Shawn J Cokus, Matteo Pellegrini

**Affiliations:** 1 Department of Molecular, Cell, and Developmental Biology, University of California, Los Angeles, CA, USA

## Abstract

**Background:**

Bisulfite sequencing using next generation sequencers yields genome-wide measurements of DNA methylation at single nucleotide resolution. Traditional aligners are not designed for mapping bisulfite-treated reads, where the unmethylated Cs are converted to Ts. We have developed BS Seeker, an approach that converts the genome to a three-letter alphabet and uses Bowtie to align bisulfite-treated reads to a reference genome. It uses sequence tags to reduce mapping ambiguity. Post-processing of the alignments removes non-unique and low-quality mappings.

**Results:**

We tested our aligner on synthetic data, a bisulfite-converted *Arabidopsis *library, and human libraries generated from two different experimental protocols. We evaluated the performance of our approach and compared it to other bisulfite aligners. The results demonstrate that among the aligners tested, BS Seeker is more versatile and faster. When mapping to the human genome, BS Seeker generates alignments significantly faster than RMAP and BSMAP. Furthermore, BS Seeker is the only alignment tool that can explicitly account for tags which are generated by certain library construction protocols.

**Conclusions:**

BS Seeker provides fast and accurate mapping of bisulfite-converted reads. It can work with BS reads generated from the two different experimental protocols, and is able to efficiently map reads to large mammalian genomes. The Python program is freely available at http://pellegrini.mcdb.ucla.edu/BS_Seeker/BS_Seeker.html.

## Background

Epigenetic regulation, such as cytosine (C) DNA methylation, is important in gene regulation and transposon silencing. The gold standard technique for studying DNA methylation is genomic bisulfite sequencing [1]. Sodium bisulfite converts unmethylated Cs to uracils, but 5-methylcytosines remain unconverted. Hence, after PCR amplification, unmethylated Cs are converted to thymines (T) while methylated Cs are unchanged. Recently, Cokus et al. and Lister et al. developed protocols, BS-Seq [2] and MethylC-seq [3], which couple bisulfite sequencing with next generation sequencing and completed a first single nucleotide resolution map of methylation in *Arabidopsis*. While these approaches open up new avenues for genome-wide measurements of DNA methylation [3-6], aligning millions of bisulfite-treated short reads (BS reads) onto the reference genome remains a challenge. Mapping bisulfite-converted reads leads to ambiguity, since Ts in the read can map to both genomic Cs or Ts. Most alignment tools, such as BLAT [7], SOAP [8], and Bowtie [9] do not explicitly enable bisulfite mapping.

Currently, there are only a few aligners explicitly designed for mapping BS reads. CokusAlignment [2] treats each cycle in a read as probabilities of A, C, T, G and uses a suffix tree searching algorithm. However, to date only the *Arabidopsis *suffix tree has been published. Other newly-developed bisulfite mapping software includes BSMAP [10], RMAP [11] and MAQ [12], which, unlike CokusAlignment, model reads as discrete base calls instead of probability vectors. BSMAP enumerates all possible combinations of C/T conversion in the BS read to find the uniquely mapping position with the least mismatches on the reference genome. It is reported [10] to have a similar sensitivity as CokusAlignment and outperformed the methods described in [3] and [4]. The bisulfite mapping in RMAP uses Wildcard matching for mapping Ts. MAQ also has a methylation alignment mode and it assigns non-unique reads randomly to one of the best-matching positions.

**Figure 1:**
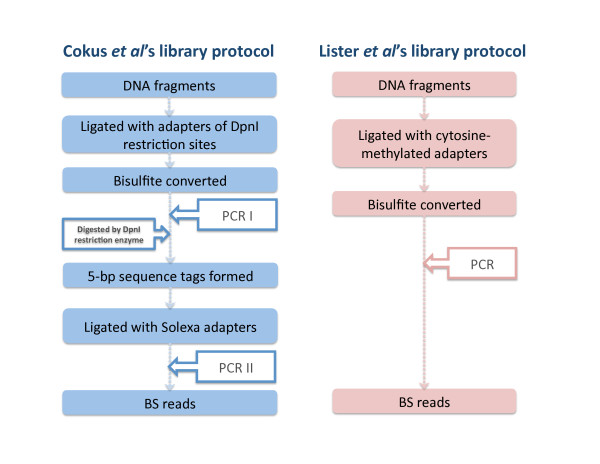
**The two library protocols generating bisulfite-converted reads**. Cokus et al's experimental protocol uses two amplification steps for generating bisulfite-converted sequences and for high throughput sequencing. The bisulfite-converted reads are preceded by one of two tags in the first 5 nucleotides of reads. Lister et al's protocol generates bisulfite libraries using premethylated adapters, and in this case no tags are present.

**Figure 2:**
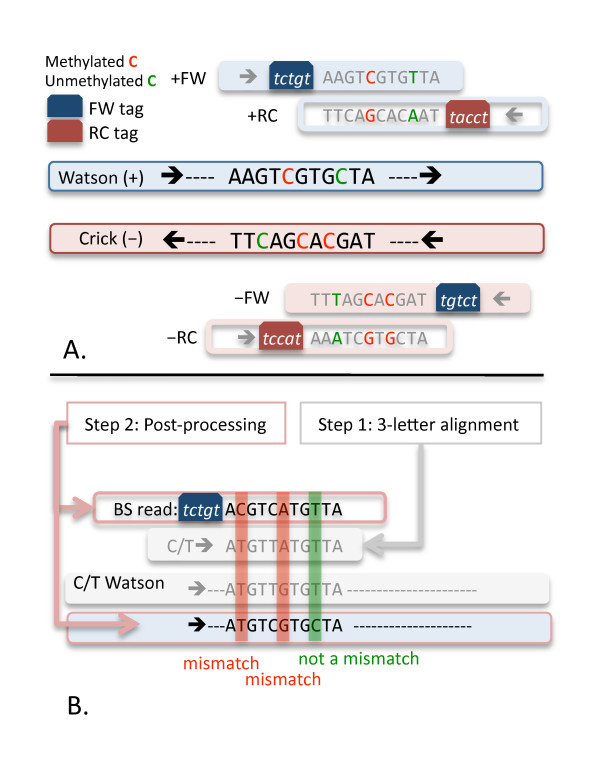
**Schematic diagrams of the 4 forms of BS reads, mapping and post processing**. 2**A**. BS reads may be in one of the 4 forms: +FW, +RC, -FW, -RC. 2**B**. Bowtie aligns C/T converted reads to the C/T converted strands. During the post processing, the number of mismatches is counted except those between read Ts and genomic Cs. Low-quality mappings with many mismatches are removed.

Two library protocols have been developed for constructing bisulfite-converted libraries (see Fig 1). Cokus et al's protocol [2] uses two amplification steps: the first amplification generates both forward and reverse bisulfite-converted sequences ligated with DNA adapters of DpnI restriction sites. These sequences are then digested by DpnI restriction enzymes that result in the 5-bp sequence tags on the bisulfite-converted sequences. There are two patterns of tags based on the forward (+FW) and reverse (-FW) directions of the bisulfite-converted sequences. After the ligation with standard Solexa adapters and the second amplification step, four types of bisulfite-converted reads are generated. They are forward and reverse reads from Watson (+FW, +RC) and Crick stands (-FW, -RC), respectively (see Fig 2A). These tags are essential to reduce the ambiguity of certain classes of reads. Unlike BSMAP, MAQ or RMAP, BS Seeker is able to explicitly use the tags to improve mapping. The second experimental protocol (i.e. Lister et al) generates bisulfite libraries using premethylated adapters, and in this case no tags are present and all reads are +FW or -FW.

BS Seeker uses Bowtie for mapping BS reads generated from either experimental protocol. It maps C/T converted FW reads to the C/T converted reference strands, and G/A converted RC reads to G/A converted, reverse complements of the reference strands. Post-processing removes low-quality mappings based on the number of mismatches. The strategy of bisulfite converting the reference sequence by treating all Cs as Ts (Gs as As) has also been used in [4,6,13], although implementations of this approach have not been published. However, most of these approaches are less precise than the ones we present here as they cannot correctly handle reads that are partially methylated.

For the evaluation of our aligner, we use synthetic BS reads to assess the accuracy of mapping. We evaluate the mapping results by comparing the methylation statistics in the synthetic reads and in the mapped sequences. We also use BS Seeker to align an *Arabidopsis *library and human libraries from the two experimental protocols, which provides us with an evaluation using real data.

## Implementation

Depending on the bisulfite experimental protocol that was used to generate the library [2,3], BS reads may be observed in either four or two forms. Cokus et al's protocol generates a forward read (+FW) from the Watson strand, the reverse complement (+RC) of +FW, a forward read (-FW) from the Crick strand, and the reverse complement (-RC) of -FW, see Fig 2A. Lister et al's protocol generates only +FW and -FW reads. We first describe how BS Seeker handles data generated from Cokus et al's protocol. It first converts all Cs to Ts on FW reads and both strands of the reference genome, so that the subsequent mapping is performed using only 3 letters, A, T, G. Similarly, G/A conversion is performed on RC reads and both strands of the reverse complement of the reference genome. Then it uses Bowtie to map the C/T converted FW reads to the C/T converted Watson and Crick strands, and the G/A converted RC reads to the two G/A converted reverse complements of the Watson and Crick strands. Reads that do not have a tag are treated as if they could be both FW and RC reads. During each of the four runs of Bowtie, the mapped positions for each read are recorded. After all the runs of Bowtie are complete, only unique alignments are retained. Here, we define unique alignments as those that have no other hits with - the same or fewer mismatches in the 3-letter alignment (between the converted read and the converted genomic sequence). Finally, we calculate the number of mismatches. For this calculation we consider a read T that aligns to a genomic C as a match, while a read C that aligns to a genomic T is considered a mismatch (see Fig 2B). Similarly, when aligning RC reads, a read A that aligns to a genomic G is considered a match, while a read G that aligns to a genomic A is considered a mismatch. Low-quality alignments with the number of mismatches larger than the user-defined value are discarded. Aligning reads generated from Lister et al's protocol is simpler, since there are no RC reads, and consequently Bowtie is only run twice.

## Results and Discussion

We first tested BS Seeker against three bisulfite aligners, BSMAP, RMAP and MAQ, by mapping synthetic reads. The sensitivity and the specificity of the aligner's output are assessed by calculating the percentage of reads that mapped uniquely and their accuracy, which is the ratio of the number of correctly mapped reads over the total uniquely mapped reads. We also calculate the inferred average methylation rate in order to discern possible mapping bias from the aligners. We then show our mapability on a lane of experimental data from *Arabidopsis*. Finally, we mapped the BS reads of human libraries generated from the two experimental protocols, in order to measure the performance of BS-Seeker on different protocols, and compare the speed of alignment to RMAP.

### Mapping synthetic reads

We simulated 36-mer BS reads from human chromosome 21. One million contained no base calling errors and another one million had base calling errors that follow the error distribution from [14] (see Supplementary Information in Additional file 1 for details). These were mapped to chromosome 21 using all four aligners. The simulated data were generated using both protocols. As shown in Table 1 and Supplementary Table S1 in Additional file 1, we found that in all cases BSMAP was significantly slower than the other aligners. MAQ's strategy, randomly assigning one of the best-matching positions for non-unique reads, results in a lower accuracy and biased estimates of the methylation rates. When the simulated reads are based on Lister et al's protocol with no tags, RMAP has a slight speed advantage over BS Seeker, but the two methods are otherwise quite similar. However, when the Cokus et al library protocol is used, BS Seeker had a higher accuracy and shorter run time than RMAP, since it is able to explicitly account for the FW/RC tags. Furthermore, the methylation rate inferred from the mapped reads using BS Seeker is very close to that of the initial synthetic data, indicating that the alignments are less biased than those of RMAP. These results suggest that BS Seeker is able to work with data from both protocols. By explicitly using tag information, it is able to optimally align reads generated by the Cokus et al. protocol.

**Table 1:** Mapping 1M synthetic human chr. 21 reads onto human chr. 21

Aligner	Experimental protocol	**Uniquely (or best **^**a**^**) mapped reads **^**b**^**(%)**	Accuracy (%)	**Methylation rates **^**c **^**(CG/CHG/CHH) (%)**			CPU time
*No base calling error*							

BS Seeker	Lister et al	91.7	100	72.0	0	0	209 s ^d^

BSMAP	Lister et al	92.1	100	72.3	0	0	15h43m20s
RMAP	Lister et al	91.7	100	72.0	0	0	185 s
MAQ	Lister et al	>99.9	93.4	67.7	0	0	353 s

BS Seeker	Cokus et al	89.6	100	72.0	0	0	263 s ^d^
BSMAP	Cokus et al	89.8	99.6	72.4	0	0	15 h 46 m 40 s
RMAP	Cokus et al	80.2	99.0	71.3	0	0.1	400 s
MAQ	Cokus et al	73.0	92.2	68.2	0	0	665 s

*Simulated base calling errors*							

BS Seeker	Lister et al	91.2	99.54	71.5	0.4	0.4	217 s
BSMAP	Lister et al	91.1	99.57	72.1	0.4	0.4	15 h 19 m 51 s
RMAP	Lister et al	91.0	99.52	71.6	0.4	0.4	188 s
MAQ	Lister et al	99.5	92.9	67.8	0.4	0.4	340 s

^a ^BS Seeker, BSMAP, and RMAP discard non-uniquely mapped reads. MAQ keeps non-uniquely mapped reads and assigns them to one of the best-matching positions. ^b ^Up to 2 mismatches are allowed. ^c ^The simulated methylation rates are set to be 72%, 0%, and 0% for CG, CHG, and CHH. ^d ^Preprocessing reference genome needs 2-5 cpu minutes for the first run.

### Mapping BS reads from *Arabidopsis*

We mapped 2,946,339 BS reads of a single lane of an *Arabidopsis *library from Cokus, et al., (2008), which is the same library tested in Xi and Li, (2009). BS Seeker is able to uniquely map 56.3% of the reads, which compares favorably to the coverage reported in Xi et al. The methylation rates inferred from the mapped reads for CG, CHG and CHH (H stands for A or C or T) are 25.5%, 8%, and 2.2% respectively, which are very close to the published results [2], i.e, 24% CG, 6.7% CHG and 1.7% CHH methylation.

### Mapping BS reads from *Human *libraries

In order to test the performance of BS Seeker on large genomes, we used it to map BS reads from human libraries generated from the two protocols [6]. The mapabilities of reads from Cokus et al's protocol and from Lister et al's protocol are very close (38.3% and 38.6% respectively). The CG methylation rate we obtained from the mapping of reads from Lister et al's protocol is 82.3%, which coincides with the published result of 82.7% [6] (see Supplementary Information in Additional file 1 for the distributions of methylation levels).

We also used RMAP to align the same human libraries. The running time for RMAP to map one lane of reads is between 11.9-13.7 hours, while for BS Seeker it is between 20-50 minutes. Both aligners showed close mapability, see Table 2. The significant advantage on the mapping efficiency suggests that BS Seeker is the most efficient bisulfite aligner for mapping large genomes.

**Table 2:** Comparison of mapping efficiency on mapping three lanes of human data

**Reads file **[6]	Number of reads	Uniquely mapped reads (%)		Running time	
		
		BS Seeker	RMAP	BS Seeker	RMAP
SRR019048	15,311,970	40.8	41.0	50 mins	13.70 hours
SRR019501	7,217,883	52.0	52.5	26 mins	11.94 hours
SRR019597	5,943,586	62.0	62.1	20 mins	13.45 hours

## Conclusions

We present a tool for mapping BS reads, the BS Seeker. It is simple to operate, and achieves high accuracy and coverage. We have made this tool publicly available for the community.

## Availability and requirements

• **Project name: **BS Seeker

• **Project home page: **http://pellegrini.mcdb.ucla.edu/BS_Seeker/BS_Seeker.html

• **Operating system(s): **Linux, Mac OS

• **Programming language: **Python

• **Other requirements: **Python 2.5.2 or higher, Bowtie 0.10.0 or higher

• **License: **Free for all users

• **Any restrictions to use by non-academics: **None, the software is readily available to any scientist wishing to use it for non-commercial purposes.

• The software (source code) and the examples are attached in Additional file 2 and Additional file 3, respectively. It can also be downloaded via the project home page.

## Authors' contributions

PC wrote BS Seeker and drafted the manuscript. SC and MP participated in the design and helped to draft the manuscript. All authors read and approved the final manuscript.

## Supplementary Material

Additional file 1 Supplementary Information; supplementary materials to the BS Seeker project. Click here for file

Additional file 2 BS Seeker software: a compressed file containing the source code for BS Seeker. Click here for file

Additional file 3 **Examples for running BS Seeker.** This compressed file includes two example datasets (reads and the reference genome) for testing BS Seeker. A step by step instruction is included in Readme and can be also viewed from the project webpage at http://pellegrini.mcdb.ucla.edu/BS_Seeker/EXAMPLES.html. Click here for file
