# Supplementary material for: BS Seeker: precise mapping for bisulfite sequencing
Source: BMC Bioinformatics. 2010 Apr 23;11:203. doi: 10.1186/1471-2105-11-203 (PMC2871274; doi:10.1186/1471-2105-11-203)
Supplement: Additional file 1 — Supplementary Information; supplementary materials to the BS Seeker project. [file 1471-2105-11-203-S1.PDF]

BS Seeker: precise mapping for bisulfite sequencing

Pao-Yang Chen, Shawn Cokus and Matteo Pellegrini*
Department of Molecular, Cell, and Developmental Biology, University of California, Los Angeles.

Supplementary Information

1 Generating synthetic BS reads

1.1 Simulating bisulfite-treated genome

We first generate the reverse complement of the reference genome, so we have both Watson (+) and Crick (-) strands available. On each strand, the methylation status for each C is then determined. Each C may be in a CG, CHG or CHH context, where H is A, C or T. To simulate a genome with a genomic average CG methylation rate of p %, each C representing CG is assigned a random float x between 0 and 1, i.e., [0,1]. If $x < p$, this C is determined as methylated. If $x >= p$, this C is determined as unmethylated and is converted to T. Similarity, CHG and CHH methylation rates are simulated on both Watson and Crick strands.

1.2 Simulating BS reads from Cokus et al's library protocols (i.e., with tags)

With Cokus et al's library protocols, four types of BS reads may be present. They are +FW, +RC, –FW and –RC. In the paper we generated equal number of these 4 types of reads from the simulated bisulfite-converted genome. To simulate reads with a length of l bps, a random number y between l and (length of genome - l), i.e., [l, (length of genome - l)], is generated. A +FW read is the l-mer sequence on the converted Watson strand, starting from location y towards the 3' end. A +RC read is the reverse complement of the l-mer sequence on the converted Watson strand, starting from location y towards the 5' end. Similar procedures are applied to generate –FW and –RC reads. Finally, FW reads are given 5-mer FW tags at their 5' ends, and RC reads are given 5-mer RC tags at their 5' ends.

1.3 Simulating BS reads from Listers et al's library protocols (i.e., with no tags)

The procedure for generating synthetic reads with no tags is the same as those described above, except we only simulate +FW and –FW reads without adding tags.

2 Introducing base calling errors in synthetic reads

Base calling errors are introduced after the BS reads are generated. The error distribution for each cycles follows the distribution shown on Figure 4b in (Dohm, et al., 2008). From the figure, the probability of error *y* and the cycle *x*, *x* is an integer from 1 to *l* (read length), is fitted by the cubic polynomial function, *y=P(x)* = 3E-06*x*^3 - 9E-05x^2 + 0.0007^x + 0.0029 (R^2 = 0.99) as shown in Figure S1.

Let s(*x*) be the nucleotide (A, C, T, or G) of the *x*-th position from the 5' end of a read, and s'(*x*)={A, T ,C, G}/{ s(*x*) }. For each cycle *x* in a read, we generate a random variable *r* between 0 and 1. If *r* < *P(x),* a nucleotide is randomly selected from *s'(x)* to replace *s(x).* If *r* >= *P(x), s(x)* stays unchanged. By moving *x* from 1 to l, we are able to simulate a read with base calling errors. We apply the same procedure to all reads.

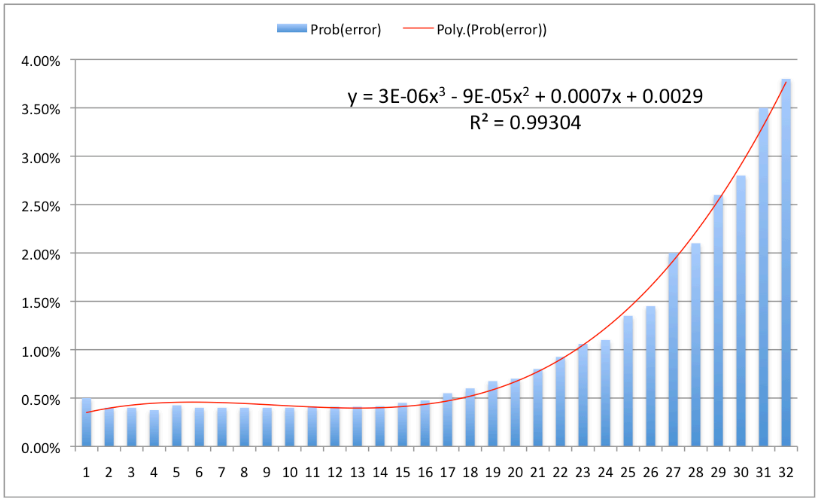

Figure S1: The error distribution per cycles fitted by a cubic polynomial

3 Bowtie parameters

Constructing the reference library

bowtie-build -f ./reference_genome/W_C2T.fa ./reference_genome/W_C2T >
./reference_genome/W_C2T.log

Mapping BS reads

bowtie -v #_mismatch --norc –k 1 --best -p 4 reference_library -f
Trimed_C2T.fa W_C2T_m#_mismatch.mapping

4 Comparison of aligners on mapping synthetic reads

The sensitivity and the specificity of the aligner's output are assessed by calculating the percentage of reads it mapped uniquely and their accuracy, which is the ratio of the number of correctly mapped reads over the total uniquely mapped reads.

Table 1 provides the results from mapping reads with no base calling errors (both protocols) and reads with base calling errors (Lister's et al's protocol).

Table S1: Mapping 1M synthetic human chr. 21 reads onto human chr. 21

Aligner	Experimental Protocol	Uniquely Mapped Readsa (%)	Accuracy (%)	Methylation ratesb (CG/CHG/CHH) (%)			CPU time (sec)
Simulated base calling errors							
BS Seeker	Cokus et al	85.2	99.5	71.5	0.5	0.5	282
BSMAP	Cokus et al	88.7	99.0	72.0	0.5	0.5	62056
RMAP	Cokus et al	81.0	98.4	70.8	0.5	0.6	400
MAQ	Cokus et al	75.1	91.4	67.6	0.5	0.5	569

5 The distributions of methylation levels from mapping two human embryonic stem cell lines

We used BS Seeker to re-map the reads generated from Lister's protocol (hESC H1 cell line, Lister et al (2009) Nature) and those from Cokus et al's protocol (hESC HSF1 cell line). The H1 map has 10x coverage, while HSF1 has only 0.6x.

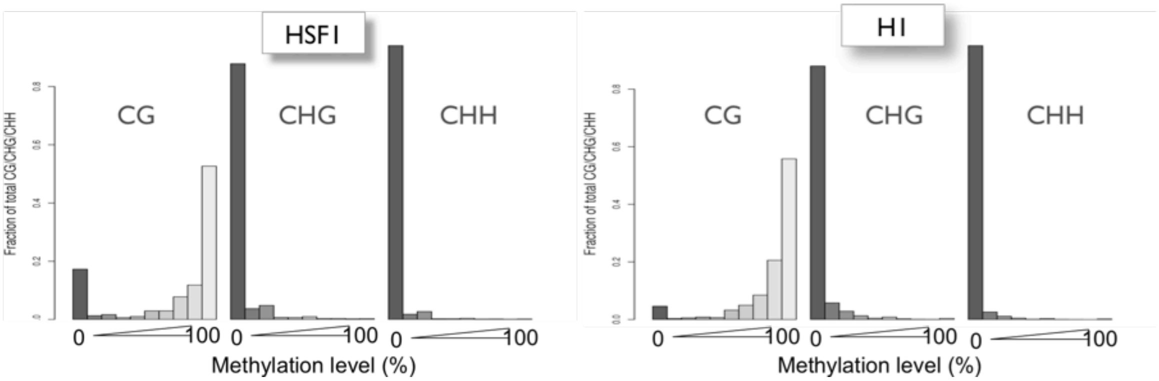

Figure S2: The distribution of methylation in H1 and HSF1 cell lines by CG, CHG, and CHH

The distributions of % methylation are quite similar in both cell lines; suggesting that the methylation rates for the hESC are not sensitive to our alignment algorithm, even when the coverage is low.

REFERENCES

Cokus, S.J., Feng, S., Zhang, X., Chen, Z., Merriman, B., Haudenschild, C.D., Pradhan, S., Nelson, S.F., Pellegrini, M. and Jacobsen, S.E. (2008) Shotgun bisulphite sequencing of the Arabidopsis genome reveals DNA methylation patterning, Nature, 452, 215-219.

Dohm, J.C., Lottaz, C., Borodina, T. and Himmelbauer, H. (2008) Substantial biases in ultra-short read data sets from high-throughput DNA sequencing, Nucleic Acids Res, 36, e105.

Xi, Y. and Li, W. (2009) BSMAP: whole genome bisulfite sequence MAPping program, BMC Bioinformatics, 10, 232.

Lister R, Pelizzola M, Dowen RH, Hawkins RD, Hon G, Tonti-Filippini J, Nery JR, Lee L, Ye Z, Ngo QM et al: Human DNA methylomes at base resolution show widespread epigenomic differences. Nature 2009, 462(7271):315-322.
